# Videomics of the Upper Aero-Digestive Tract Cancer: Deep Learning Applied to White Light and Narrow Band Imaging for Automatic Segmentation of Endoscopic Images

**DOI:** 10.3389/fonc.2022.900451

**Published:** 2022-06-01

**Authors:** Muhammad Adeel Azam, Claudio Sampieri, Alessandro Ioppi, Pietro Benzi, Giorgio Gregory Giordano, Marta De Vecchi, Valentina Campagnari, Shunlei Li, Luca Guastini, Alberto Paderno, Sara Moccia, Cesare Piazza, Leonardo S. Mattos, Giorgio Peretti

**Affiliations:** ^1^Department of Advanced Robotics, Istituto Italiano di Tecnologia, Genoa, Italy; ^2^Unit of Otorhinolaryngology – Head and Neck Surgery, IRCCS Ospedale Policlinico San Martino, Genoa, Italy; ^3^Department of Surgical Sciences and Integrated Diagnostics (DISC), University of Genoa, Genoa, Italy; ^4^Unit of Otorhinolaryngology – Head and Neck Surgery, ASST Spedali Civili of Brescia, Brescia, Italy; ^5^Department of Medical and Surgical Specialties, Radiological Sciences, and Public Health, University of Brescia, Brescia, Italy; ^6^The BioRobotics Institute and Department of Excellence in Robotics and AI, Scuola Superiore Sant’Anna, Pisa, Italy

**Keywords:** larynx cancer, oral cancer, oropharynx cancer, machine learning, endoscopy, laryngoscopy, computer vision, otorhinolaryngology

## Abstract

**Introduction:**

Narrow Band Imaging (NBI) is an endoscopic visualization technique useful for upper aero-digestive tract (UADT) cancer detection and margins evaluation. However, NBI analysis is strongly operator-dependent and requires high expertise, thus limiting its wider implementation. Recently, artificial intelligence (AI) has demonstrated potential for applications in UADT videoendoscopy. Among AI methods, deep learning algorithms, and especially convolutional neural networks (CNNs), are particularly suitable for delineating cancers on videoendoscopy. This study is aimed to develop a CNN for automatic semantic segmentation of UADT cancer on endoscopic images.

**Materials and Methods:**

A dataset of white light and NBI videoframes of laryngeal squamous cell carcinoma (LSCC) was collected and manually annotated. A novel DL segmentation model (*SegMENT)* was designed. *SegMENT* relies on *DeepLabV3+* CNN architecture, modified using *Xception* as a backbone and incorporating ensemble features from other CNNs. The performance of *SegMENT* was compared to state-of-the-art CNNs (*UNet*, *ResUNet*, and *DeepLabv3*). *SegMENT* was then validated on two external datasets of NBI images of oropharyngeal (OPSCC) and oral cavity SCC (OSCC) obtained from a previously published study. The impact of in-domain transfer learning through an ensemble technique was evaluated on the external datasets.

**Results:**

219 LSCC patients were retrospectively included in the study. A total of 683 videoframes composed the LSCC dataset, while the external validation cohorts of OPSCC and OCSCC contained 116 and 102 images. On the LSCC dataset, *SegMENT* outperformed the other DL models, obtaining the following median values: 0.68 intersection over union (IoU), 0.81 dice similarity coefficient (DSC), 0.95 recall, 0.78 precision, 0.97 accuracy. For the OCSCC and OPSCC datasets, results were superior compared to previously published data: the median performance metrics were, respectively, improved as follows: DSC=10.3% and 11.9%, recall=15.0% and 5.1%, precision=17.0% and 14.7%, accuracy=4.1% and 10.3%.

**Conclusion:**

*SegMENT* achieved promising performances, showing that automatic tumor segmentation in endoscopic images is feasible even within the highly heterogeneous and complex UADT environment. *SegMENT* outperformed the previously published results on the external validation cohorts. The model demonstrated potential for improved detection of early tumors, more precise biopsies, and better selection of resection margins.

## Introduction

At present, the cornerstone of the otolaryngological clinical examination of the upper aero-digestive tract (UADT) is represented by endoscopy. Whether performed through the nose with flexible instrumentation or transorally by rigid telescopes, endoscopy, especially if coupled with high-definition (HD) technology, provides a detailed, magnified, and comprehensively enhanced vision of the UADT. Endoscopy enhancing filters (EEFs), such as Narrow Band Imaging (NBI) or the Storz Professional Image Enhancement System (SPIES), have been playing a fundamental role in the past decade, empowering conventional white light (WL) endoscopy by highlighting the submucosal and subepithelial neoangiogenic network associated with malignant transformation (1). By enhancing visualization of the cancer-related abnormal intrapapillary capillary loops, these “bioendoscopic” tools have been shown to provide better performance compared to standard WL endoscopy in the diagnosis of UADT carcinomas (2–5). Nowadays, EEFs like NBI are widely used in various head and neck subsites such as the larynx/hypopharynx (3, 6, 7), oropharynx (8, 9), nasopharynx (10–12), and oral cavity (13–15), where they play a fundamental role in detection, characterization, and delineation of superficial margins of malignant lesions. However, caution is needed in the analysis and interpretation of UADT videoendoscopies, especially in centers less experienced with these techniques. Even with EEFs, in fact, the detection and evaluation of vascular abnormalities is limited by the considerable heterogeneity in the appearance of squamous cell carcinomas (SCCs) of this area. Moreover, margins delineation can be challenging when mucosal vascularization is altered by other factors, such as inflammatory disease or previous (chemo)radiotherapy (16). Finally, several aspects hinder the large-scale implementation of EEFs during routine UADT endoscopic assessment, such as its intrinsic operator-dependent nature and the relatively steep learning curve needed to master this technique.

Artificial intelligence (AI) is a potentially powerful ally to support clinicians in this complex scenario, prompting our research group to envision the birth of “Videomics” as a new and promising field of application of such a tool in the diagnostic challenges of the UADT oncologic diseases (17). The term Videomics was coined to refer to computer vision and deep learning methods that are systematically used to process the unstructured video data obtained from diagnostic endoscopy to convert subjective assessment into objective findings. Parallelly, the use of AI in videoendoscopy, especially in the gastrointestinal field, has already become relevant in the literature and even on the market (18). When moving to the specific field of UADT, however, only a few studies have been published in the current literature, with most focusing on laryngeal endoscopy (19). Among all AI-powered methods, deep learning (DL) techniques based on convolutional neural networks (CNNs) are increasingly used in UADT videoendoscopy analysis for automatic disease detection (20–22), classification (23, 24), and segmentation (25). In fact, thanks to their unique architecture, CNNs provide improved performance compared to conventional computer vision and machine learning methods.

Image segmentation is typically used to locate objects in images by marking their specific contours and the area inside those. In computer vision, semantic segmentation is referred as the task of assigning each pixel in an image to a predefined set of classes. Within the different computer vision tasks, semantic segmentation is particularly interesting for UADT endoscopy. Indeed, the possibility offered by DL to automatically detect tumor boundaries, especially if coupled with EEFs imaging, would represent a valuable support in clinical practice. This could make the benefits of EEFs accessible to all physicians and contribute to improve their performance in tumor recognition and margins delineation. However, only a few studies have pursued automatic segmentation of UADT lesions so far (26–29) and, thus, further research is needed to progress this technology and advance it towards its clinical implementation.

In this paper, we describe a new CNN-based semantic segmentation model for videoendoscopy of the UADT, named *SegMENT.* This model was specifically developed for the identification and segmentation of UADT cancer in endoscopic video frames, with particular attention to laryngeal squamous cell carcinoma (LSCC), oral cavity squamous cell carcinoma (OCSCC) and oropharyngeal squamous cell carcinoma (OPSCC). The list of abbreviations used in the article is reported in **Table 1**.

**Table 1:** Abbreviations and acronyms.

AI	Artificial Intelligence
ANOVA	Analysis of Variance
ASPP	Atrous Spatial Pyramid Pooling
CNN	convolutional neural network
DL	Deep Learning
DSC	Dice Similarity Coefficient
EEF	Endoscopy Enhancing Filter
FPS	Frames Per Second
FN	False Negative
FP	False Positive
HD	High-Definition
IoU	Intersection over Union
LSCC	Laryngeal Squamous Cell Carcinoma
NBI	Narrow Band Imaging
OPSCC	Oropharyngeal Squamous Cell Carcinoma
OCSCC	Oral Cavity Squamous Cell Carcinoma
SPIES	Storz Professional Image Enhancement System
SCC	Squamous Cell Carcinoma
TP	True Positive
TN	True Negative
TL	Transfer Learning
UADT	Upper Aero-Digestive Tract
VIA	VGG Image Annotator
WL	White Light

## Materials and Methods

### Data Acquisition

Recorded videoendoscopies of patients treated between 2014 and 2019 at the Unit of Otorhinolaryngology – Head and Neck Surgery of the IRCSS Ospedale Policlinico San Martino, Genoa, Italy, were retrospectively revised. Selection criteria comprised a pathology report positive for LSCC and the availability of at least one recorded videoendoscopy before treatment. Local Institutional Review Board approval was obtained (CER Liguria: 230/2019). All patients were first examined through transnasal videolaryngoscopy (HD Video Rhino-laryngoscope Olympus ENF-VH, Olympus Medical System Corporation, Tokyo, Japan) in the office before treatment. For those submitted to transoral laryngeal microsurgery, an additional intraoperative endoscopic evaluation was conducted using 0°, 30° or 70° telescopes coupled to a HD camera head connected to a Visera Elite CLV-S190 light source (Olympus Medical System Corporation). In both settings, a thorough examination was conducted under WL videoendoscopy, then switching to NBI.

**Figure 1:**
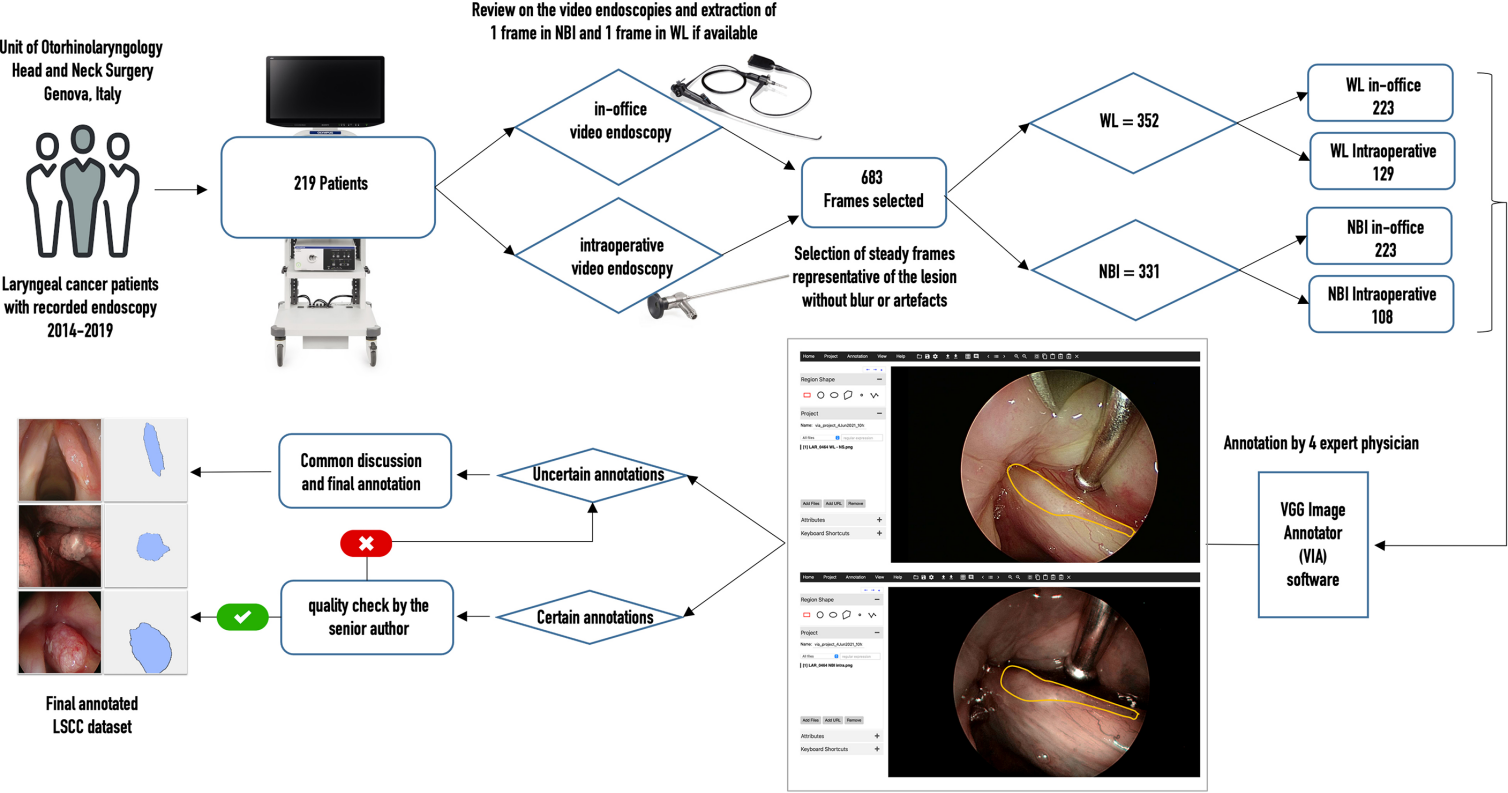
Flow chart of data acquisition for the laryngeal squamous cell carcinoma (LSCC) dataset. WL, white light; NBI, narrow band imaging.

From each of the collected videos, four expert physicians extracted, when available, one WL and one NBI frame. Frames were selected to be the most representative of the lesion and possibly offer a clear view of its boundaries. Priority in image selection was given to steady frames with few artifacts and blur. The extracted videoframes were then labeled by the same physicians using the VGG Image Annotator (VIA) 2.0 (https://www.robots.ox.ac.uk/~vgg/software/via/), an open-source web-based annotation software. The annotation process consisted in the manual segmentation of the neoplastic lesion borders: this was done by manually tracing its contour following the visible tumor margins identified both in WL and in NBI. The pixels comprised in the traced regions were classified as “LSCC” and no specific label was assigned for NBI or WL images. If multiple lesions were visible, multiple segmentations were carried out in order to select all the LSCC pixels in the image. If a physician was not completely sure about the correctness of the annotations, all four otolaryngologists revised them collectively. Finally, a senior surgeon (the author G.P.) checked all annotations and referred the inexact ones for collective revision. **Figure 1** summarizes the data acquisition process.

Finally, two datasets of NBI endoscopic images were obtained from a previous study on automatic segmentation of OCSCC and OPSCC (26). The datasets included the corresponding ground-truth annotations, as previously described.

### *SegMENT* Architecture

**Figure 2:**
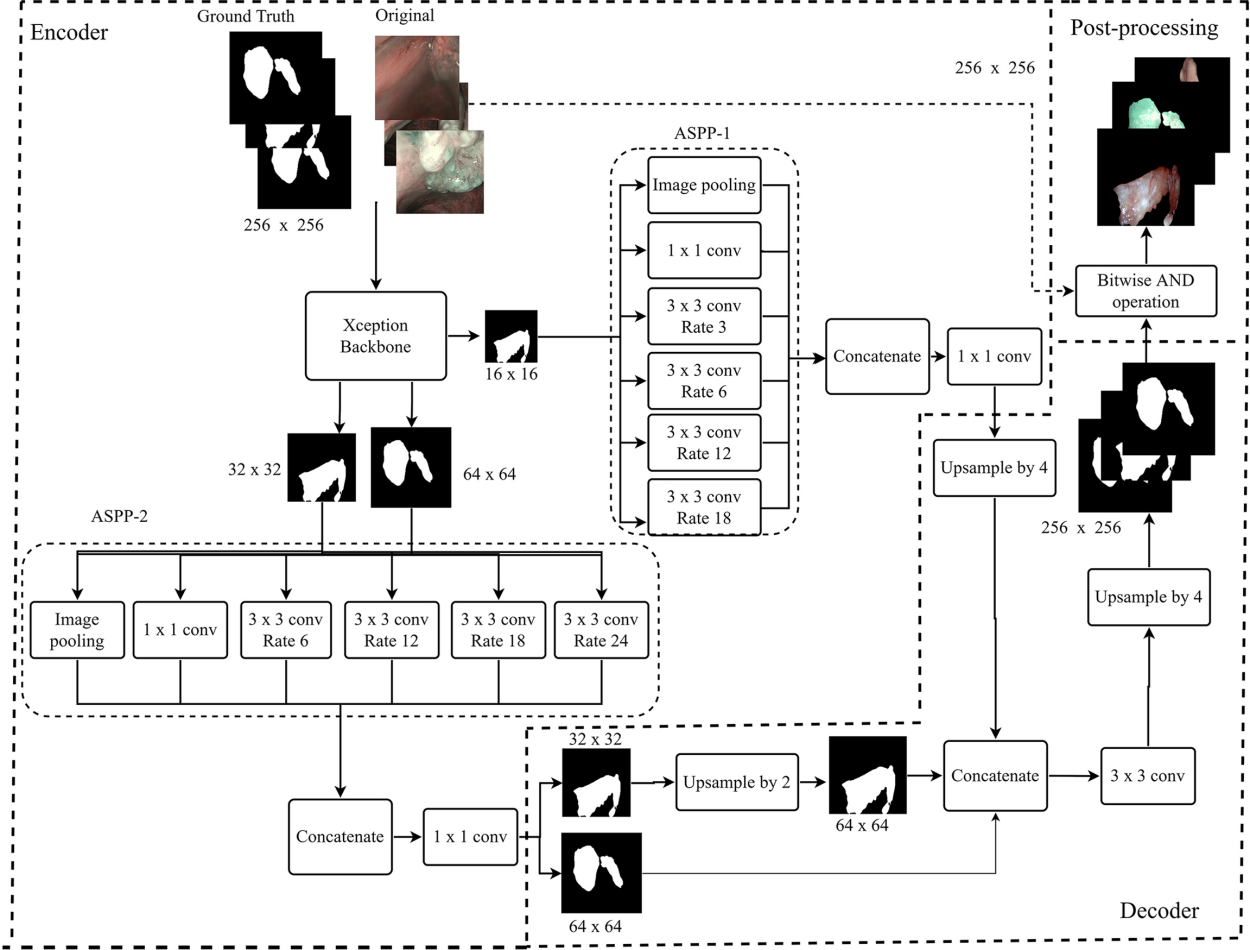
Workflow diagram of the proposed *SegMENT* semantic segmentation architecture. The *Xception* backbone is customized for segmentation tasks by eliminating the dense layers for classification while maintaining the feature extraction layers. The model encoder uses two ASPP blocks designed, respectively, for high- and low-level image features. The decoder section concatenates the information coming from the two ASPP blocks, producing segmentation masks.

**Figure 2** describes the architecture of *SegMENT*. This latter is based on concepts introduced by the *DeepLabV3+* model (30), which were expanded and customized here for precise cancer segmentation in UADT videoendoscopies. *The segMENT* backbone was built on the *Xception* architecture (31), which was chosen for its high benchmark results on ImageNet (32), the largest dataset of natural images publicly available. *Xception* has a smaller number of parameters compared to the most popular CNN architectures like *VGG16* and *VGG19*, but an almost equal number of parameters than *Resnet50* and *DensNet121* (33). The *Xception* backbone architecture is composed of two primary components: convolutional layers with pooling for feature extraction, and fully connected dense layers at the top of the network for classification. To customize this backbone network for segmentation tasks, we removed the network fully connected dense layers and maintained only the feature extraction layers. The functionalities to resize the input frames into 256x256 pixels were maintained. In addition, we used three-skip connections to get feature map outputs (of size 16×16, 32×32, and 64×64 pixels) from *Xception* backbone convolution layers. These were then merged using Atrous Spatial Pyramid Pooling (ASPP) blocks in the encoder part of our model.

Given the heterogeneous nature of UADT lesions in terms of dimension, form, and contour, we designed the encoder part of *SegMENT* to use two ASPP blocks. These can potentially contribute to increased segmentation accuracy. The ASPP block-1 is designed for high-level image features (shapes, tumor composition, etc.). It is fed with 16×16 pixels input images directly from the *Xception* backbone and contains 256 filters. ASPP block-1 comprises five rates of dilation convolution layers (1, 3, 6, 12, and 18), which were chosen given the small-scale input images. The ASPP block-2 is designed for low-level image features (edges, contours, texture) and accepts two scales of input images (32×32 and 64×64 pixels). It is also comprised of five rates of dilation convolution layers (1, 6, 12, 18, and 24), which are higher here because of the larger scale of the input images. The ASPP block-2 employs 48 filters for 64×64 pixels input images and 64 filters for 32×32 pixels input images. Finally, the convolutional layers of *SegMENT* use the Mish activation function (except for the last layers that use a sigmoid activation function). This activation function was selected to replace the traditional Relu activation function as it was shown to provide better performance (34). The decoder section accepts the encoder outputs with the three resized (16×16, 32×32, and 64×64 pixels) images. The output images of ASPP block-1 are four times up-sampled using the UpSampling2D layer and bilinear interpolation technique to yield 64×64 pixels images. The images from ASPP block-2 with 32×32 pixels are up-sampled two times. Next, 64×64 pixel images from ASPP block-2 are concatenated with the up-sampled 64×64 pixel images generated by ASPP block-1. This concatenation produces 64×64 pixels images. Afterward, a 2D convolutional layer with a kernel size of 3×3 and 256 filters is applied to these images. Again, images are further up-sampled four times to get 256×256 pixel images. Finally, the segmented tumor area is retrieved through a bitwise-AND operation, which generates an output image of 256x256 pixels.

### Baseline Models

Three state-of-the-art baseline CNNs for semantic segmentation (i.e., *UNet, ResUNet, and DeepLabv3+*) were investigated and tested as part of a comparative study. Each segmentation model incorporates a backbone architecture for feature extraction. The backbones considered were *VGG16, VGG19, MobileNetV2, ResNet50, ResNet101V2, DenseNet121*, and *Xception*. Pre-trained backbone weights from the ImageNet dataset were used (32). The *UNet* network (35) is equipped with an encoding path that learns to encode texture descriptors and a decoding path that achieves the segmentation task. *ResUNet* (36) is a segmentation model based on the *UNet* architecture that implements residual units instead of plain neural units, in order to obtain good performance with fewer parameters. Finally, *DeepLabV3+* (30) uses dilated separable convolutions and spatial pyramid pooling in a U-shaped architecture to produce accelerated inference times and reduced loss values. The training and testing of these baseline models were performed in the same environment and using the same data as *SegMENT*.

### Ensemble Technique

**Figure 3:**
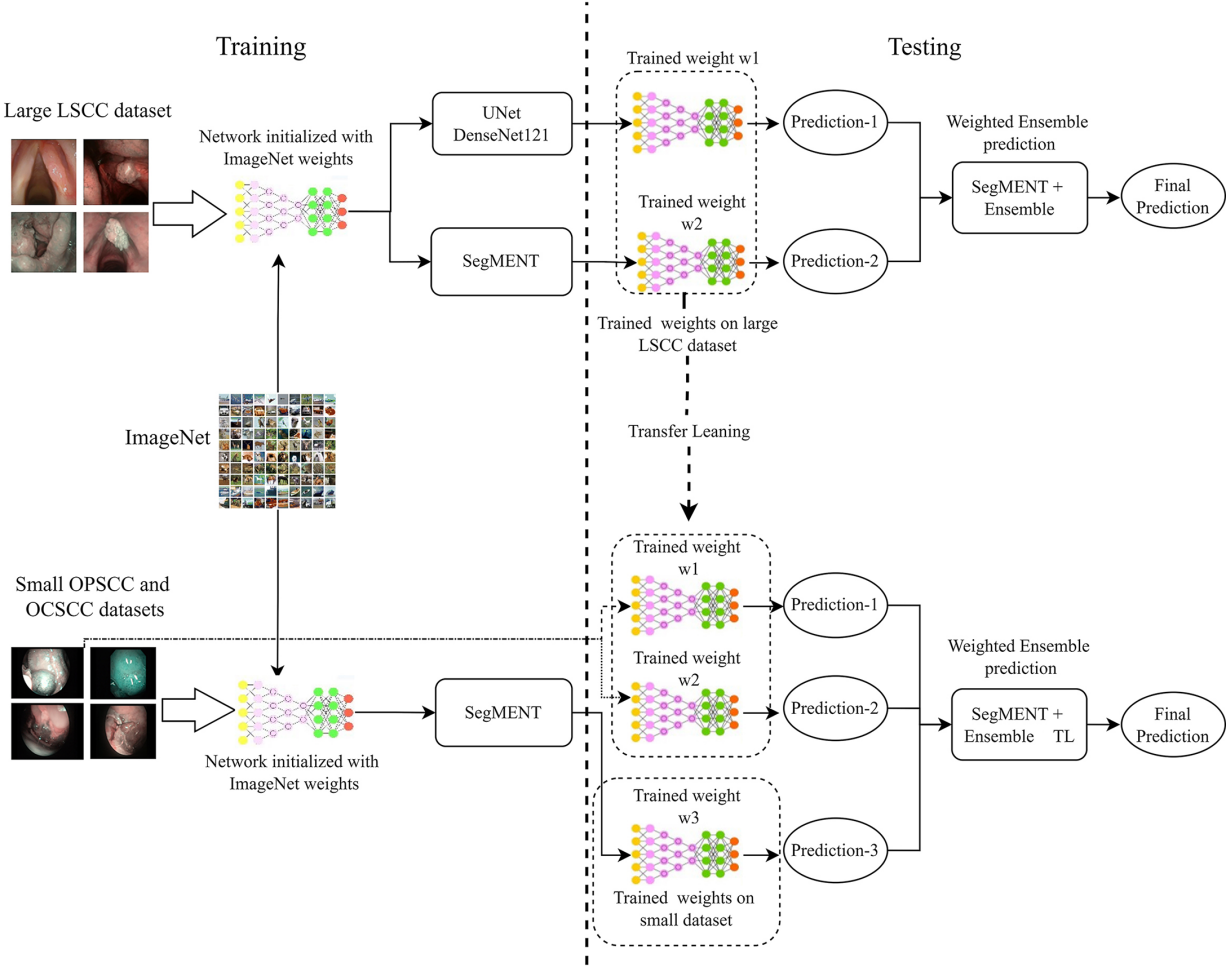
Diagram describing the training and testing of the models assessed in this work. Initially, the models are initialized with weights obtained from training on the ImageNet dataset. Next, the models are trained on the specific datasets of interest. During testing, the trained models provide segmentation predictions, and ensemble used to generate the final segmentations. In addition, in-domain transfer learning (TL) can be used to enhance the segmentation performance on small datasets using trained weights from other anatomical subsites. LSCC, laryngeal squamous cell carcinoma; OCSCC, oral cavity squamous cell carcinoma; OPSCC, oropharyngeal squamous cell carcinoma.

Multiple ensemble techniques are described in the literature for decreasing segmentation errors and optimizing efficiency (37–39). Their utility becomes evident especially when the available training dataset for a new application area is small or highly heterogeneous, such as the case of the OCSCC and OPSCC datasets. In this work, we evaluated the value of using the weighted average ensemble approach (27) during the testing phase of the segmentation network. This technique implements a weighted ensemble of predictions from different models. Its integration into the proposed segmentation architecture is shown in **Figure 3**.

In LSCC segmentation, the predictions of the two best-performing models (*SegMENT* and *UNet-DenseNet121*) were combined to improve the accuracy of laryngeal cancer segmentation. Initially, the two models were independently trained on the LSCC dataset. Their predictions were then combined following the weighted average ensemble approach.

In OCSCC segmentation tasks, three different predictions were ensembled during testing. One was taken from a *SegMENT* model trained on the OCSCC dataset. The other two predictions were taken from *SegMENT* and *UNet-DenseNet121* trained on the LSCC dataset. To produce the single final output prediction, the three output predictions were multiplied by the assigned weight values, which were obtained through a grid search technique.

The same strategy was used for OPSCC segmentation, with the difference that the first *SegMENT* model was trained on the OPSCC dataset. The other two predictions were taken from *SegMENT* and *UNet-DenseNet121* models trained on the LSCC dataset, as before.

### Training Parameters

For training of *SegMENT*, the Tverky loss function (40) was used. This loss function is used for highly unbalanced datasets. In our model, we used a learning rate of 0.001 with a batch size of 8 images per epoch during training. The learning rate decay was set to a factor of 0.1. If the training loss did not improve after four consecutive epochs of learning, the decay was slowed down. Data augmentation was used during training to increase the variability of the training dataset: flip, crop, translation, rotation, and scaling were applied, as well as hue, brightness, and contrast augmentation. A Tesla K80 GPU with 12 GB of memory and an Intel(R) Xeon(R) CPU running at 2.20 GHz with 13 GB of memory using Keras and a Tensorflow (41) back-end were used for all experiments.

### Validation on LSCC Dataset

The first experiments were performed on the LSCC dataset, which was split into a training and a test sets using a respective split ratio of 80% and 20% with randomly selected images. Initially, the baseline models and *SegMENT* were trained and tested on the LSCC dataset starting from pre-trained weights obtained from ImageNet. Afterward, the weights of *SegMENT* and *UNet-DenseNet121* were combined using the weighted ensemble method in the testing phase.

### Validation on OCSCC and OPSCC Datasets

The OCSCC and OPSCC datasets were separately used to validate *SegMENT* on these different UADT sites. Each dataset was split into a training and a validation/test groups with a 70/30 percent split ratio based on a random image selection process.

SegMENT was first trained and tested separately on the OCSCC and OPSCC datasets, starting from ImageNet pre-trained weights without applying the ensemble technique. Following this, the described in-domain transfer learning (TL) method based on a weighted ensemble technique was used to assess potential performance improvements. We hypothesized that, compared to the standard TL provided by ImageNet, which is based on natural images (such as daily objects, and animals), a specific in-domain TL based on LSCC trained weights might enable better performance on images from other UADT regions. Therefore, the *SegMENT* ensemble model, incorporating features initially learned from the LSCC dataset, was tested on the OCSCC and OPSCC datasets during the testing phase of the segmentation framework.

### Outcome Analysis

The outcomes of each DL model were evaluated by comparing the predicted segmentations with the manual annotations performed by expert physicians (i.e., the ground-truth segmentations). Standard evaluation metrics for semantic segmentation were used as previously reported (42). A classification of each pixel in the images as true positive (TP), true negative (TN), false positive (FP), or false negative (FN) was used to derive the evaluation metrics below.

- *Accuracy*: the percentage of pixels in the image that is correctly classified by the model.

$$Accuracy=\frac{\text{TP}+\text{TN}}{\text{TP}+\text{FP}+\text{TN}+\text{FN}}$$

- *Precision* (positive predictive value): the fraction of pixels that are true positives (correctly predicted pixels of the targeting class) among the total predicted pixels:

$$Precision=\frac{\text{TP}}{\text{TP}+\text{FP}}$$

- *Recall* (sensitivity): the fraction of pixels that are true positives among the total ground truth segmented pixels:

$$Recall=\frac{\text{TP}}{\text{TP}+\text{FN}}$$

- *Dice similarity coefficient* (DSC): represent the harmonic weight of Precision and Recall values (also called F1 score):

$$DSC=\frac{2\text{TP}}{2\text{TP}+\text{FN}+\text{FP}}$$

**Figure 4:**
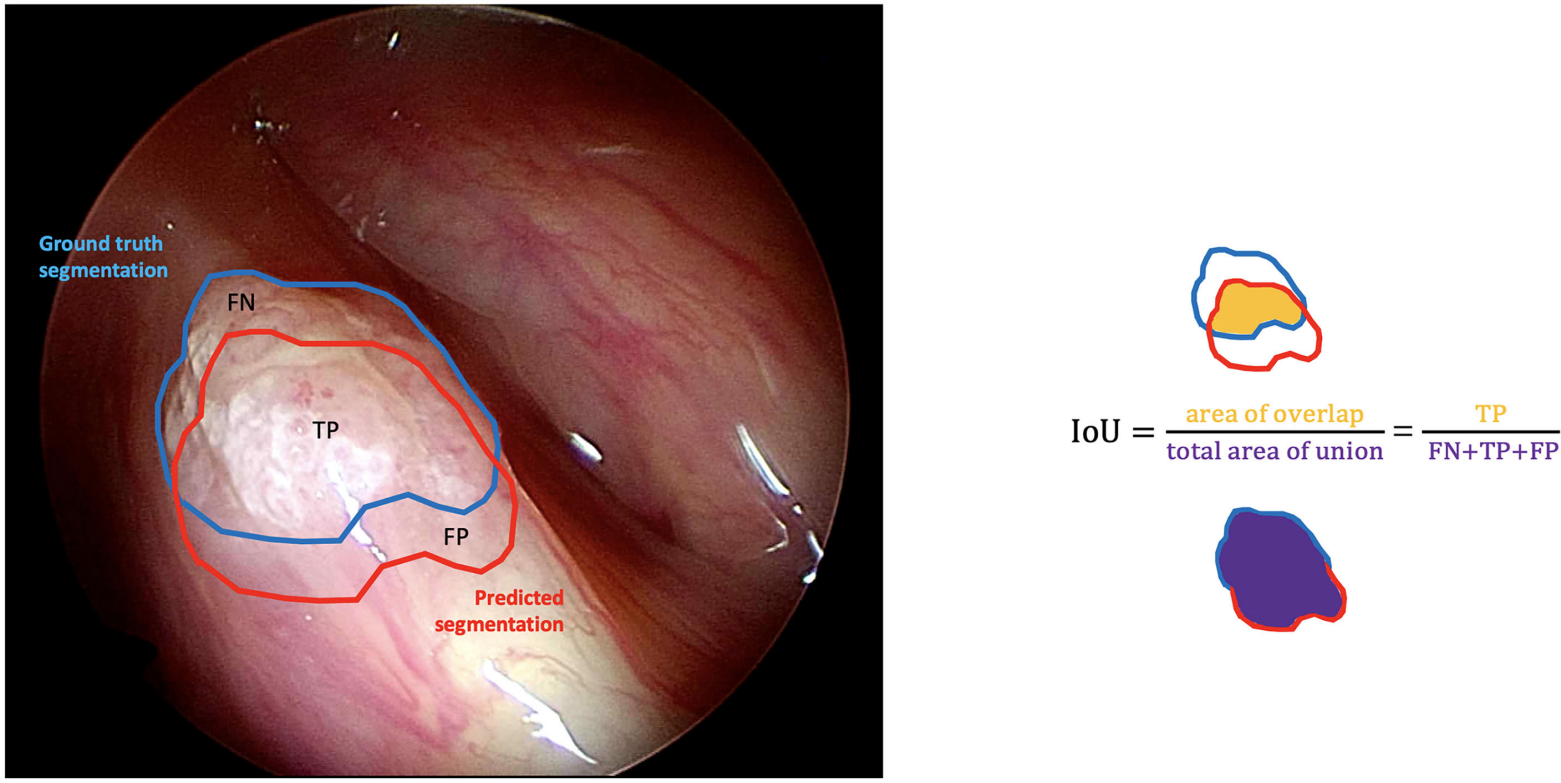
Graphical representation of the intersection over union (IoU) calculation on a white light right glottic cancer intraoperative videoframe. The boundary traced in light blue represents the ground truth segmentation provided by an expert clinician, while the red one represents the model prediction. The IoU is calculated by dividing the overlapping area (containing the true positive pixels) by the total area of union (encompassing the false negative, true positive, and false positive pixels).


- *Intersection over Union* (IoU): the fraction of pixels that are true positives among the union of pixels that are positive predictions and belong to the target class (**Figure 4**).

$$loU=\frac{\text{TP}}{\text{TP}+\text{FN}+\text{FP}}$$

### Statistical Analysis

Differences in distributions of continuous variables among more than two independent groups were assessed with the analysis of variance (ANOVA) test. *Post-hoc* analysis was performed using Tukey’s multiple comparisons test to control for the inflated Type I error. A p<0.05 was considered significant. Data analysis was carried out using statistical functions (scipy.stat) and statistical models (statmodels v0.13.2) libraries in python (v3.9).

## Results

**Figure 5:**
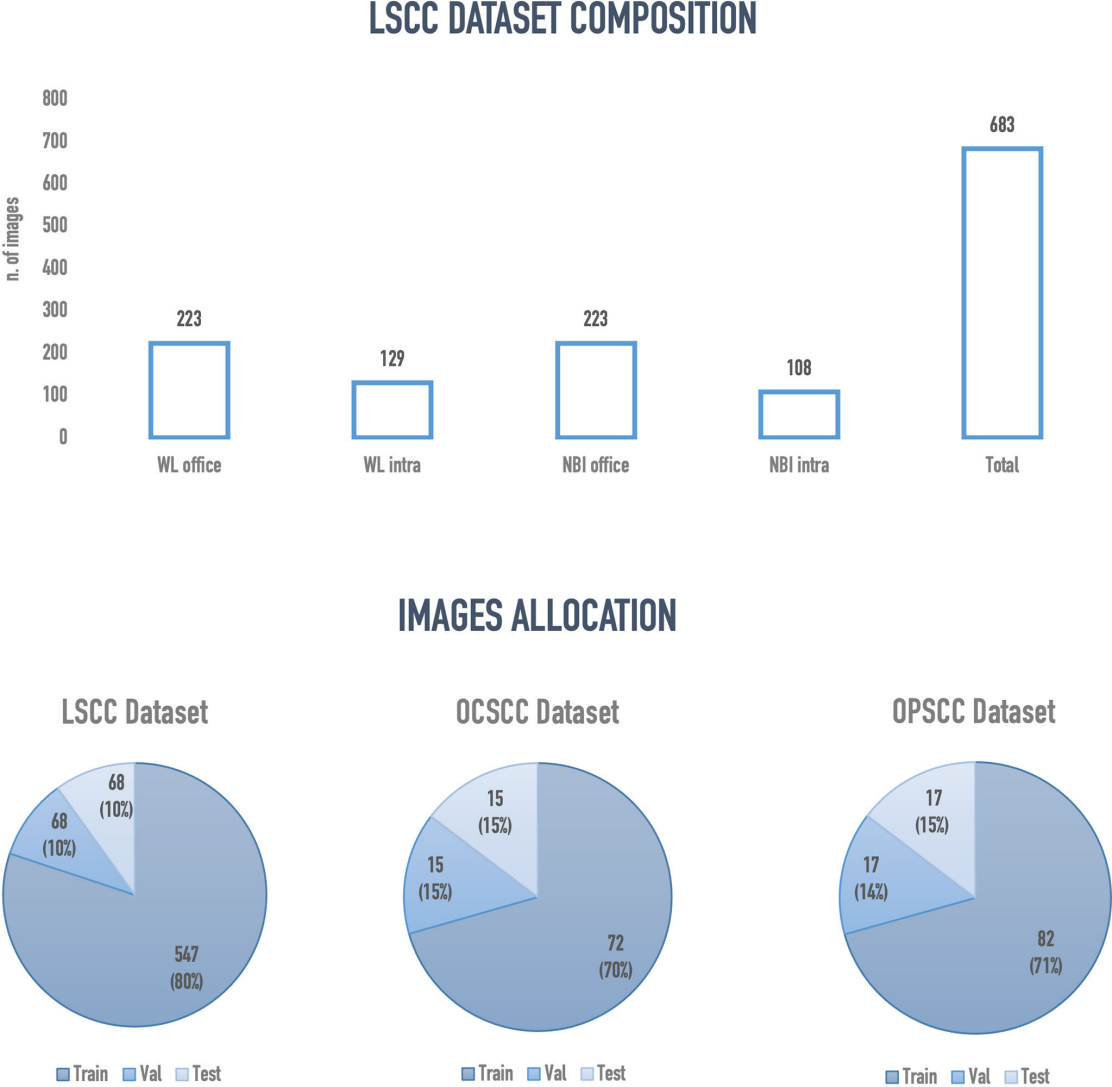
Overview of the final configuration of laryngeal squamous cell carcinoma (LSCC), oral cavity squamous cell carcinoma (OCSCC) and oropharyngeal squamous cell carcinoma (OPSCC) dataset. Val, validation; WL, white light; NBI, narrow band imaging; Intra, intraoperative endoscopy acquired image; Office, inoffice endoscopy acquired image.

Two hundred and nineteen patients with a mean age of 67.9 years (SD ± 11.8 years) were enrolled. Among these, 196 (89.4%) were males and 23 (10.6%) females. A total of 683 frames representing LSCC were extracted from videolaryngoscopies. Of those, 223 were in-office WL, 129 intraoperative WL, 223 in-office NBI, and 108 intraoperative NBI images. **Figure 5** presents an overview of the final composition of the LSCC dataset.

**Figure 6:**
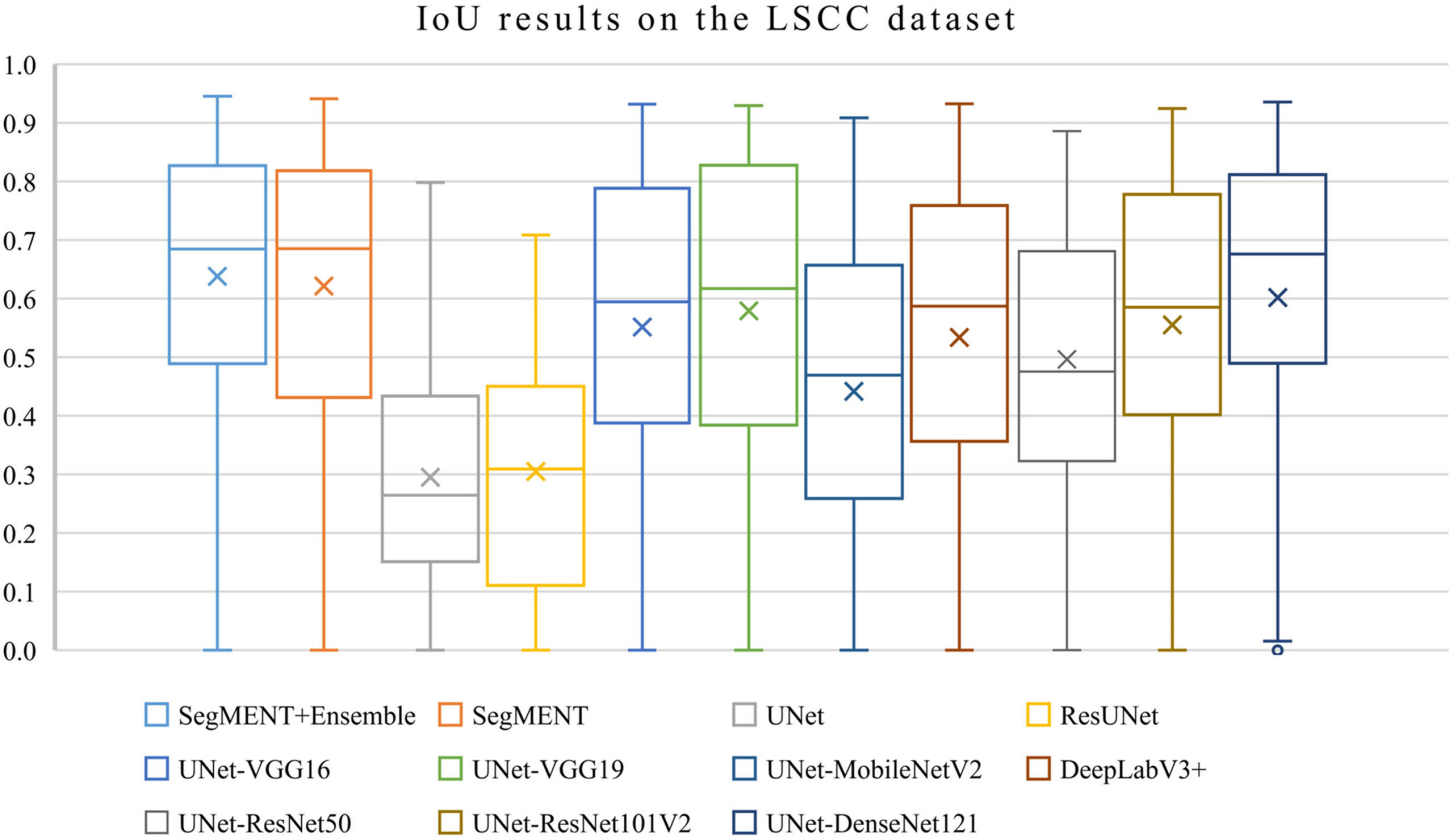
Boxplots of Intersection over Union (IoU) results from *SegMENT* ensemble and the other state-of-the-art segmentation models on the laryngeal squamous cell carcinoma (LSCC) dataset. The cross sign represents the mean value while the horizontal line inside the boxplot shows the median value.

**Figure 7:**
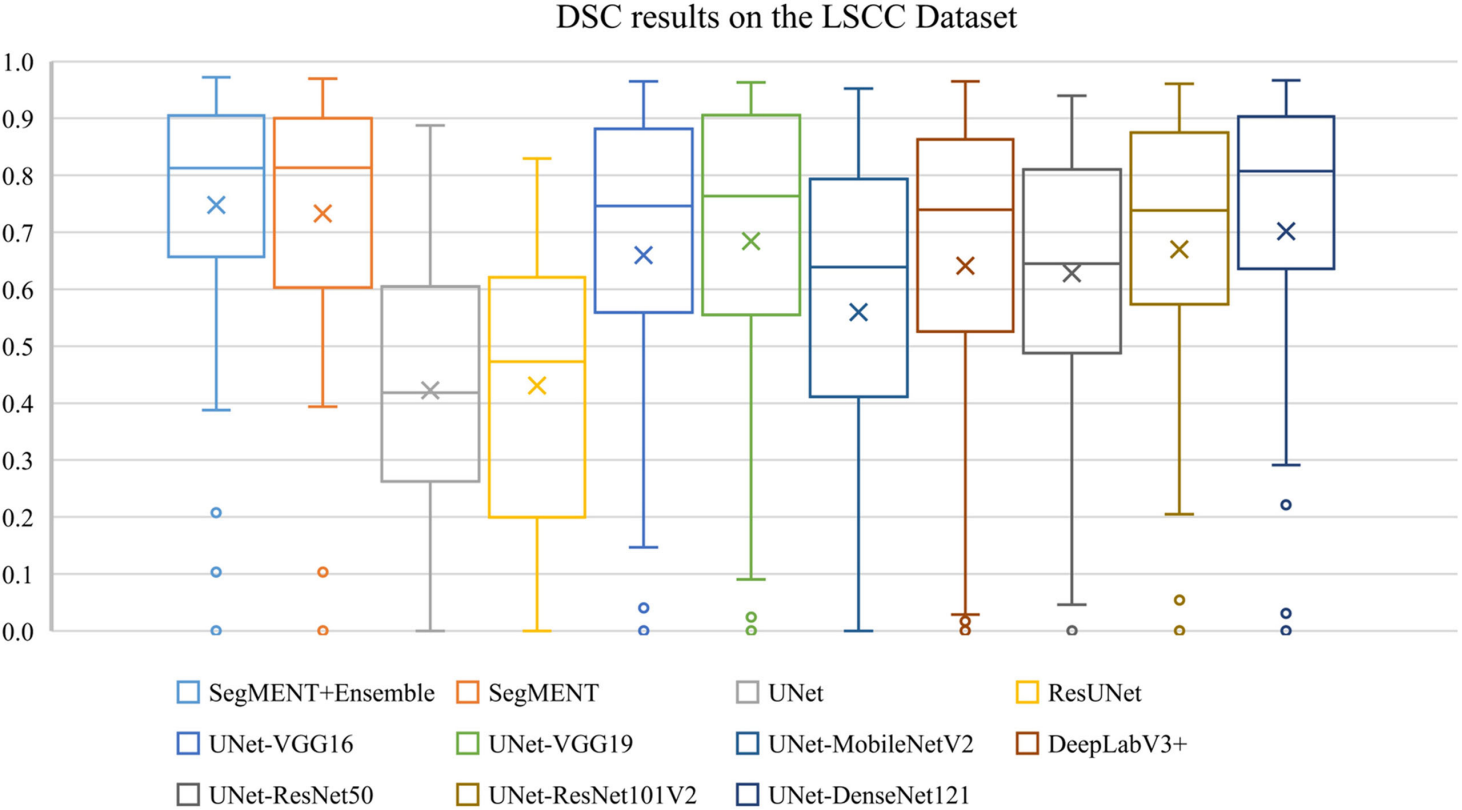
Boxplots of Dice similarity coefficient (DSC) results from *SegMENT* ensemble and the other state-of-the-art segmentation models on the laryngeal squamous cell carcinoma (LSCC) dataset. The cross sign represents the mean value, while the horizontal line inside the boxplot shows the median value.

**Figure 8:**
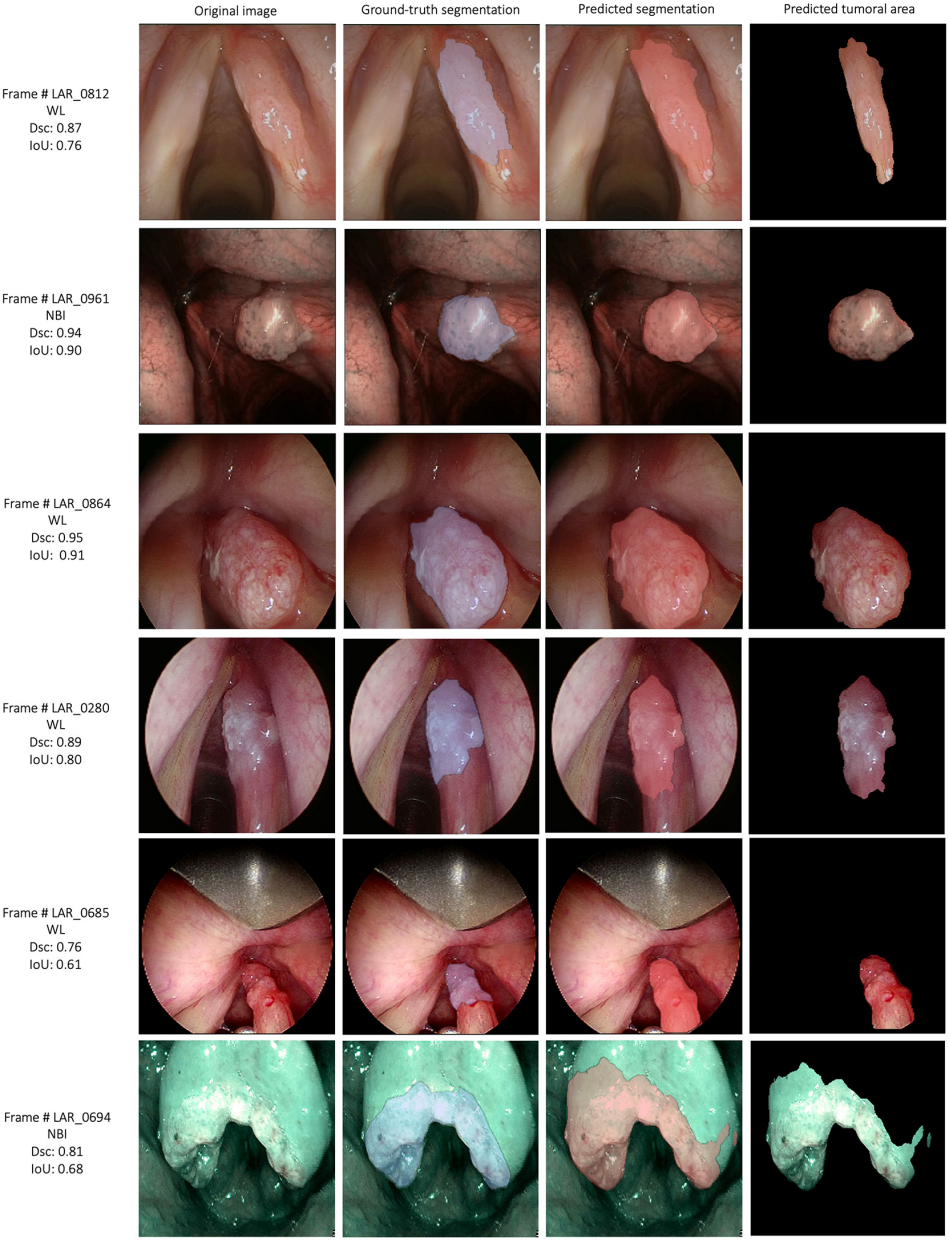
Examples of automatic segmentation results for the laryngeal squamous cell carcinoma dataset using *SegMENT* ensemble. DSC, dice similarity coefficient; IoU, intersection over union; WL, white light; NBI, Narrow Band Imaging.

The semantic segmentation models were trained on the LSCC dataset after random distribution of the images into a training set (547 images), a validation set (68 images), and a test set (68 images). During experiments, it was observed that the proposed *SegMENT* model outperformed the other state-of-the-art segmentation models. Among the baseline models, the *UNet-DenseNet121* performed better than the other baseline models. Thus, the ensemble technique was used to integrate the training weights from this model with those from *SegMENT*, leading to better segmentation performance during testing. The performances of the models on the test set are shown in **Table 2**. The median values achieved by *SegMENT* with the ensemble technique on the LSCC dataset were: IoU=0.685, DSC=0.814, recall=0.951, precision=0.785, and accuracy=0.973. The boxplots showing the IoU and DSC score performances of *SegMENT* and the other state-of-the-art segmentation models during testing on the LSCC dataset are shown in **Figures 6**, **7**. The processing rates of all base-line models ranged from 6.3 to 8.7 frames per second (fps), while the proposed ensemble model processed an average of 2.1 fps (taking 0.48 seconds to process a single frame). Examples of LSCC segmentation including ground-truth labels and the resulting automatic segmentations are shown in **Figure 8**.

**Table 2:** Performance evaluation of different semantic segmentation models during testing on the laryngeal squamous cell carcinoma (LSCC) dataset.

Dataset	Model	Backbone	IoU	DSC	Recall	Precision	Accuracy
Larynx Cancer (LSCC)	UNet	–	0.264	0.418	0.641	0.351	0.895
	ResUNet	–	0.309	0.473	0.486	0.490	0.928
	UNet	VGG16	0.595	0.746	0.817	0.823	0.968
	UNet	VGG19	0.618	0.763	0.900	0.827	0.967
	UNet	MobileNetV2	0.469	0.639	0.579	**0.855**	0.961
	DeepLabV3+	ResNet50	0.587	0.740	0.714	0.830	0.968
	UNet	ResNet50	0.476	0.645	0.834	0.745	0.952
	UNet	ResNet101V2	0.586	0.739	0.841	0.788	0.963
	UNet	DenseNet121	0.677	0.807	0.847	0.840	0.971
	SegMENT	Xception	**0.686**	**0.814**	0.916	0.830	0.969
	SegMENT ensemble	Xception	0.685	**0.814**	**0.951**	0.785	**0.973**

Values in bold denote the best results. IoU, intersection over union; DSC, dice similarity coefficient. The results represent the median scores from all the tests for each metric.

When comparing results among all the models using ANOVA test, the differences were significant for each metric (p<0.001). When performing multiple comparisons on IoU, the *SegMENT* ensemble model achieved significantly better results than *UNet*, *ResUNet*, *UNet-MobileNetV2*, and *UNet-ResNet5*0 CNNs (p=0.001, p=0.001, p=0.001, and p=0.03, respectively). Concerning DSC, the *SegMENT* ensemble model achieved a significantly better result compared to *UNet*, *ResUNet*, and *UNet-MobileNetV2* CNNs (p=0.001, p=0.001, and p=0.001, respectively). Considering recall, the *SegMENT* ensemble model significantly outperformed *UNet*, *ResUNet*, *UNet-VGG16*, *UNet-MobileNetV2*, and *DeepLabv3+*CNNs (p=0.001, p=0.001, p=0.02, p=0.001, and p=0.001, respectively). For precision and accuracy values, the *SegMENT* ensemble model performed significantly better compared to *UNet* and *ResUNet* (p=0.001 and p=0.001, respectively).

**Figure 9:**
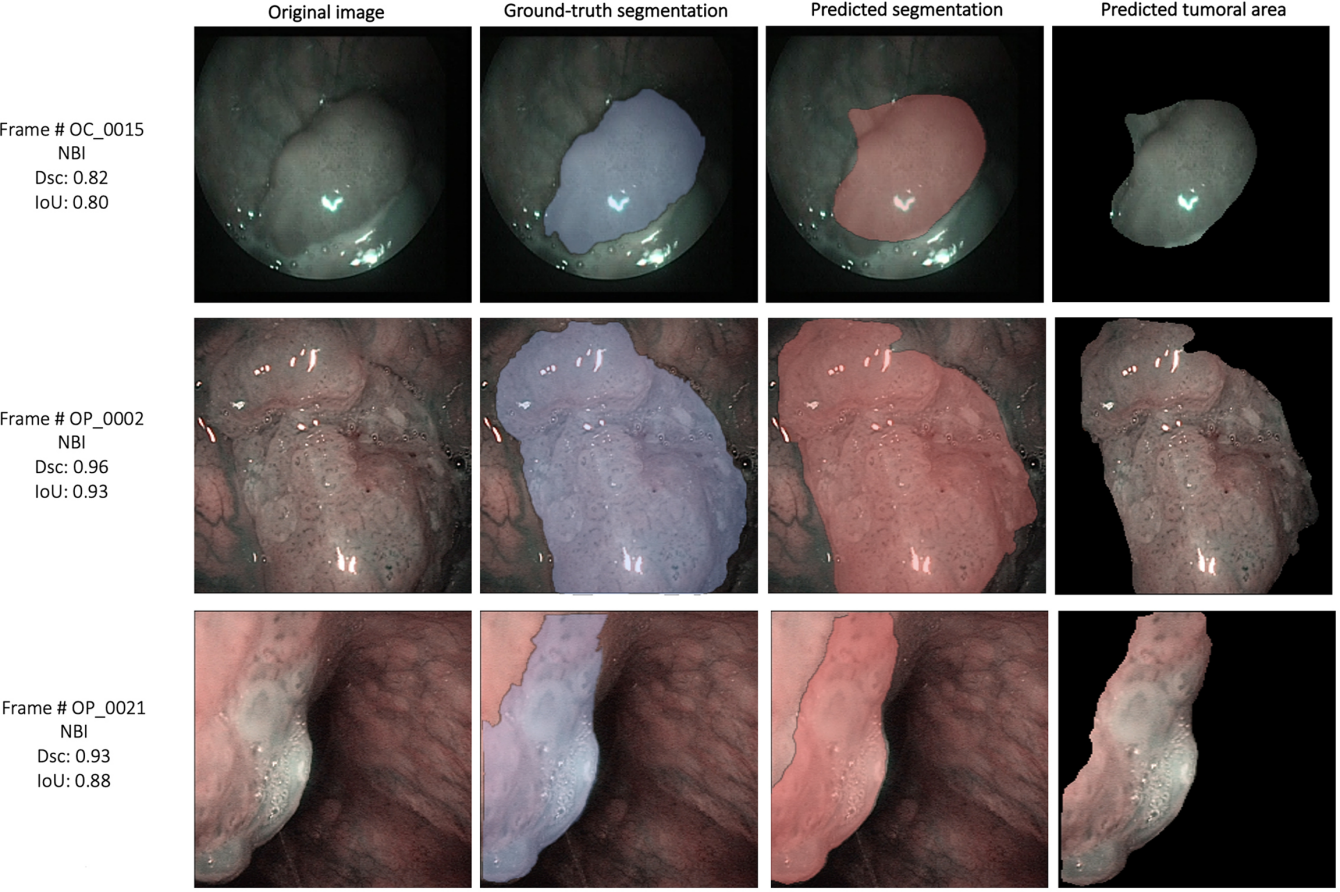
Examples of automatic segmentation results for the oral cavity and oropharyngeal squamous cell carcinoma datasets using *SegMENT* + ensemble in-domain transfer learning. DSC, Dice similarity coefficient; IoU, intersection over union; NBI, Narrow Band Imaging.

The external validation cohorts comprised 102 images for OCSCC (72 for training, 15 for validation, and 15 for testing) and 116 images for OPSCC (82 for training, 17 for validation, and 17 for testing). Previously published outcomes (26) are compared to the proposed models performance in **Table 3**. However, while the images used were the same, it must be highlighted that it was not possible to perform the same exact image allocation in the training/testing cohorts as in the previous study. The *SegMENT* model pre-trained on ImageNet already performed better than the previous study CNNs on all metrics. The in-domain TL also helped to improve the results, especially for the OPSCC dataset. Indeed, the median metrics on the OCSCC and OPSCC datasets improved compared to the previously published by, respectively, 10.3% and 11.9% for DSC, 15.0% and 5.1% for recall, 17.0% and 14.7% for precision, and 4.1% and 10.3% for accuracy. The processing rate of our model was 3.9 fps on both the OCSCC and OPSCC datasets. Examples of segmentation of OCSCC and OPSCC frames displaying both the ground-truth labels and the resulting automatic segmentations are shown in **Figure 9**.

**Table 3:** Performance evaluation of models during testing on the oral cavity (OCSCC) and oropharynx squamous cell carcinoma (OPSCC) datasets.

Dataset	Model	Backbone	IoU	DSC	Recall	Precision	Accuracy
Oral Cavity Cancer (OCSCC)	UNet (26)	–	–	0.654	0.755	0.632	0.890
	ResNet with 5×2 blocks (26)	–	–	0.656	0.670	0.708	0.879
	SegMENT	Xception	0.612	**0.759**	0.757	**0.878**	**0.931**
	SegMENT + ensemble TL	Xception	**0.749**	0.598	**0.905**	0.602	0.917
Oropharynx Cancer (OPSCC)	UNet (26)	–	–	0.712	0.815	0.704	0.819
	ResNet with 4×2 blocks (26)	–	–	0.760	0.856	0.772	0.830
	SegMENT	Xception	0.685	0.786	0.767	0.874	0.932
	SegMENT + ensemble TL	Xception	**0.784**	**0.879**	**0.907**	**0.919**	**0.933**

The results achieved by *SegMENT* trained only on the specific datasets and by the *SegMENT* ensemble model (i.e., incorporating features learned from the LSCC dataset) are compared to those reported employing UNet and ResNet in the previous study (26). The results represent the median scores from all the tests for each metric. Values in bold denote the best results. IoU, intersection over Union; DSC, dice similarity coefficient; TL, transfer learning.

## Discussion

The development of semantic segmentation AI models for medical image analysis is a field of study that is becoming increasingly widespread, especially for radiologic imaging (43–45). Conversely, the exploitation of these algorithms to investigate videoendoscopic images represents a sphere of research less explored in the literature, as demonstrated by the lack of proper terminology to indicate such a field of interest before our first proposal to identify it as “Videomics” (17). Notably, the vast majority of studies analyzing the applicability of DL-based semantic segmentation models in endoscopy come from the gastrointestinal field (46, 47). On the other hand, reports regarding the use of such algorithms in videoendoscopic examination of the UADT are scarce. This can be attributed to many factors: first, the insidious and diversified mucosal anatomy and a wide variety of lesions arising from this region are a challenge for AI-based image recognition models; second, while HD colonoscopy and esophago-gastroduodenoscopy have been routinely used in worldwide screening protocols for decades (48, 49), implementation of UADT endoscopic examination coupled with HD-videoendoscopy is more recent and less widespread, thus limiting the amount of data available for the application of AI technologies.

In this study, the authors present the promising results of a new CNN specifically designed for the automatic segmentation of UADT SCC. This new processing network was first tested on a dataset of LSCC images and subsequently validated on OCSCC and OPSCC images obtained from a previous study (26). The proposed model showed similar diagnostic outcomes for all three investigated sites, demonstrating good generalization capacity and, thus, the potential for using it in real-life clinical scenarios. Considering the tests performed on the LSCC dataset, the *SegMENT* ensemble model performed better than the state-of-the-art CNNs, especially considering the IoU and Dsc metrics, which are the most reliable and widely used for the evaluation of semantic segmentation models. These performances were maintained when the model was validated on the OCSCC and OPSCC cohorts, where SegMENT outperformed the results of the state-of-the-art models previously investigated on the same datasets (26). Notably, the adjunction of specific-TL features borrowed from the LSCC dataset allowed reaching even higher results, especially for the OPSCC dataset, compared to the basal *SegMENT* pre-trained on ImageNet. While most methods for medical image analysis employ TL from general natural images (e.g., ImageNet) (32) this strategy has been proven to be less effective compared to in-domain TL due to the mismatch in learned features between natural and medical images (50). Similarly, our results indicate that in-domain TL is a promising strategy for the processing of UADT videoendoscopies. Nonetheless, our findings should be further validated on larger datasets, as the results on the OCSCC dataset were improved less compared to OPSCC. These contradictory outcomes may be explained by the heterogeneous image composition of the OCSCC dataset. Indeed, endoscopic examination of the oral cavity often includes videoframes of the lip cutaneous surfaces, alveolar ridges, or teeth crowns. Therefore, these non-mucosal areas that differ markedly from the laryngeal and oropharyngeal endoscopic appearance may have contributed to confuse the model and decrease its performance. Moreover, the limited number of images included in the validation datasets might limit the effect of in-domain TL which we believe could lead to even better results if tested on more images.

The comparable results obtained by the proposed model for the LSCC (WL+NBI), the OCSCC (NBI only), and OPSCC (NBI only) datasets confirm the good generalization capacity of this model, which performs well regardless of the light source used to acquire images. Moreover, even without in-domain TL, the *SegMENT* ensemble model maintained its performance on the OCSCC and OPSCC datasets regardless of the small training datasets (13% and 15%, respectively, compared to the LSCC dataset). This finding possibly suggests that EEFs images, with their enhanced visual characteristics, may offer more information to the model during the training phase, thus helping to mitigate the shortage of images. Nevertheless, a prospective study comparing WL vs. EEFs cohorts is recommended to better investigate this finding.

To date, the present study represents the first attempt to validate a DL-based semantic segmentation model capable of achieving good results in the endoscopic assessment of SCC arising from the oral cavity, oropharynx, and larynx. The segmentation task has been seldomly applied to videoendoscopic images of the UADT, making this field of study innovative. Laves *et al.* previously tested different CNNs to automatically delineate different tissues and anatomical subsites on images obtained during intraoperative endoscopic evaluation of the glottis (27). The authors reported high mean values of IoU (84.7%) by segmenting every object in the image but did not focus on the annotation of cancer. Moreover, their dataset consisted of similar images obtained from only two patients, hence impairing a reasonable comparison with our results. A more specific paper on laryngeal lesion segmentation was published by Ji and colleagues (28). In this work, several CNN models were implemented to delineate glottic leucoplakias on a dataset of 649 images with segmentation metrics in line with our results (DSC=0.78 and IoU=0.66). Interestingly, the best processing performance achieved by their models was 5 fps which, together with the previous results of Paderno and colleagues (ranging within 8.7 and 16.9 fps) (26), were faster than the 3.9 fps processed by our proposed model. Notwithstanding, all these processing times are still far from real-time inferences (20-30 fps), meaning that different strategies and different CNNs must be explored in order to maintain high diagnostic performance while reaching real-time efficiency. Investigating a different UADT subsite, Li and colleagues employed a CNN to automatically segment endoscopic images of nasopharyngeal carcinoma (29). Their work included a large dataset of WL in-office endoscopic frames (30,396 images) obtained in a single tertiary-level institution. Of note, the only segmentation metric reported is mean DSC (0.75±0.26), which is comparable to the results of our model. Interestingly, the authors underlined the value of the proposed semantic segmentation algorithm as an instrument to perform a target biopsy in case of suspicious nasopharyngeal lesions. Indeed, the common presence of adenoid/lymphatic hyperplasia in this area burdened the performance of endoscopic biopsies taken in an office-based setting, thus resulting in considerable false negatives rates (29). The same issue is frequently encountered when performing endoscopy-driven biopsies of suspicious neoplasms of other UADT districts. Regarding the oropharynx, lesions arising from the base of tongue and amygdalo-glossal sulcus are frequently hidden by lymphatic tissue present in this area, and can sometimes be misinterpreted and confused with mucosal/lymphatic hyperplasia (26). Similarly, tumors in the oral cavity can nest in inflammatory lesions such as leucoplakias or lichen, which may also hinder the real target to biopsy. Regarding the larynx, the performance of incisional biopsies under WL has been largely questioned due to its low sensitivity (51). The introduction of EEFs, by allowing to better select the most suspicious area to target, represented an important step forward leading to a significant improvement in the performances of endoscopic biopsies (2, 52–54). Nevertheless, the difficulties encountered during human evaluation of such images burden the capillary application of EEFs, hence paving the way to the innovative application of AI for these tasks. Indeed, the use of trustworthy semantic segmentation DL models during endoscopy may automatically delineate the superficial area where to conduct the biopsy, even when facing heterogeneous and mystifying lesions such as SCCs of the UADT.

Additionally, automatic-segmentation models may find a field of application even intraoperatively for surgical guidance (55), or for driving tumor excision and provide an improved rate of negative surgical margins. Of note, the use of NBI during surgery for SCC of the oral cavity, oropharynx, and larynx has been already shown to be effective in these tasks (56–59), but we believe that AI-based tools, once rigorously validated, will represent a more precise and objective method for surgical margins sampling. Finally, pursuing the automatic segmentation in this field is expected to become increasingly relevant in the future not only for surgical practice but in other fields as well. In fact, semantic segmentation is paramount for establishing boundaries between objects in order to explain complex situations to computers. In the future, highly elaborated AI tools might be able to autonomously understand the relationships between different elements, even in a highly complex environment such as the UADT, and suggest meaningful clinical decisions to physicians.

The present work has limitations that must be acknowledged. First, the study conclusions are restricted by the small size of the datasets, especially the external validation cohorts, and by its retrospective design. To overcome such drawbacks, an enriched data collection will characterize our future projects in order to increase the dataset’s size. Additionally, data acquisition protocols will be implemented by gathering videos from different video sources, with the purpose of enhancing the generalization capability of the algorithm. Furthermore, the previously collected NBI images from the validation cohorts did not allow head-to-head comparison of WL vs. NBI. Moreover, as *SegMENT* was trained to recognize the appearance of lesions, the finding of diffuse and narrow intrapapillary capillary loops, typical of inflammatory diseases or radiotherapy, may lead to decreased lesion segmentation performance. To minimize this potential issue, future research should consider training CNN models using endoscopic images obtained from heterogeneous cohorts of patients, including those that were previously irradiated or are concomitantly affected by inflammatory diseases. Finally, the annotations performed by physicians were not cross-validated by other institutions, representing a bias that will be addressed in future studies.

## Conclusions

This work represents the first multicentric validation of a DL-based semantic segmentation model applied on UADT videoendoscopic images of SCC. The model maintained reliable diagnostic performance analyzing both WL and NBI images from three distinct anatomical subsites. Ensemble strategies and in-domain transfer learning techniques demonstrated the potential to increase segmentation performance. Exploration of new CNNs should be carried out to pursue real-time clinical implementation, while further studies powered by a larger training dataset and larger external validation cohorts are needed before setting up clinical trials.

## Data Availability Statement

The raw data analyzed during the current study are available from the corresponding author on reasonable request for research purposes only.

## Ethics Statement

The studies involving human participants were reviewed and approved by Ethics Committee of IRCCS Ospedale Policlinico San Martino, Genova, Italy (CER Liguria). Written informed consent for participation was not required for this study in accordance with the national legislation and the institutional requirements.

## Author Contributions

Conceptualization, MA, AI, CS, and LM; Data curation, MA, AI, CS, PB, GG, MV, VC, SL, LG, and AP; Formal analysis, MA, CS, and SL; Investigation, MA, AI, CS, PB, GG, MV, VC, and LM; Methodology, MA, AI, CS, LM, and SM; Supervision, SM, CP, GP, LG, and LM; Writing—original draft, CS, AI, MA, SM, and LM; Writing—review and editing, AI, CS, MA, SM, CP, AP, GP, and LM. All authors contributed to the article and approved the submitted version.

## Conflict of Interest

The authors declare that the research was conducted in the absence of any commercial or financial relationships that could be construed as a potential conflict of interest.

## Publisher’s Note

All claims expressed in this article are solely those of the authors and do not necessarily represent those of their affiliated organizations, or those of the publisher, the editors and the reviewers. Any product that may be evaluated in this article, or claim that may be made by its manufacturer, is not guaranteed or endorsed by the publisher.
